# Systematic review of cognitive deficits in adult mitochondrial disease

**DOI:** 10.1111/ene.14068

**Published:** 2019-10-22

**Authors:** H. L. Moore, A. P. Blain, D. M. Turnbull, G. S. Gorman

**Affiliations:** ^1^ Newcastle University Newcastle upon Tyne UK; ^2^ NIHR Newcastle BRC NuTH‐NHS Foundation Trust Newcastle upon Tyne UK

**Keywords:** adult, cognition, impairment, memory, mitochondrial disease

## Abstract

The profile and trajectory of cognitive impairment in mitochondrial disease are poorly defined. This systematic review sought to evaluate the current literature on cognition in mitochondrial disease, and to determine future research directions. A systematic review was conducted, employing PubMed, Medline, Psycinfo, Embase and Web of Science, and 360‐degree citation methods. English language papers on adult patients were included. The literature search yielded 2421 articles, of which 167 met inclusion criteria. Case reports and reviews of medical reports of patients yielded broad diagnoses of dementia, cognitive impairment and cognitive decline. In contrast, systematic investigations of cognitive functioning using detailed cognitive batteries identified focal cognitive rather than global deficits. Results were variable, but included visuospatial functioning, memory, attention, processing speed and executive functions. Conclusions from studies have been hampered by small sample sizes, variation in genotype and the breadth and depth of assessments undertaken. Comprehensive cognitive research with concurrent functional neuroimaging and physical correlates of mitochondrial disease in larger samples of well‐characterized patients may discern the aetiology and progression of cognitive deficits. These data provide insights into the pattern and trajectory of cognitive impairments, which are invaluable for clinical monitoring, health planning and clinical trial readiness.

## Introduction

Mitochondrial diseases are a common group of genetic neuromuscular disorders characterized by genotypic and phenotypic heterogeneity, with a prevalence similar to that of many other genetically determined neurodegenerative diseases 1. Although there is significant clinical variability, neurological impairment remains a hallmark of mitochondrial disease and cognitive impairment is one of the least understood aspects.

A review of clinical manifestations by El‐Hattab and colleagues 2 identified initial symptoms of developmental delay in <10% of patients and impaired mentation in 10–24% of patients referred to as having mitochondrial encephalopathy, lactic acidosis and stroke‐like episodes (MELAS). Overall, 50–74% featured learning disability or memory impairment and ≥90% experienced dementia. Prevalence rates of cognitive difficulties have ranged from 0 to 90%, depending on genotype and stage of disease 3, 4, 5, 6, 7, 8, 9, 10. These findings would indicate variable cognitive outcomes for patients with mitochondrial disease. Nevertheless, to date, our knowledge of intellectual ability in patients with mitochondrial disease and how this may change with disease progression remains scarce.

The purposes of this systematic review were twofold: (i) to investigate current literature on cognition in mitochondrial disease and (ii) to determine how the field must advance to improve our knowledge of cognition in this group.

## Methods

Throughout this systematic review, we have adhered to the Preferred Reporting Items for Systematic Reviews and Meta‐Analyses (PRISMA) guidelines for conducting systematic reviews 11.

### Search strategy

**Figure 1:**
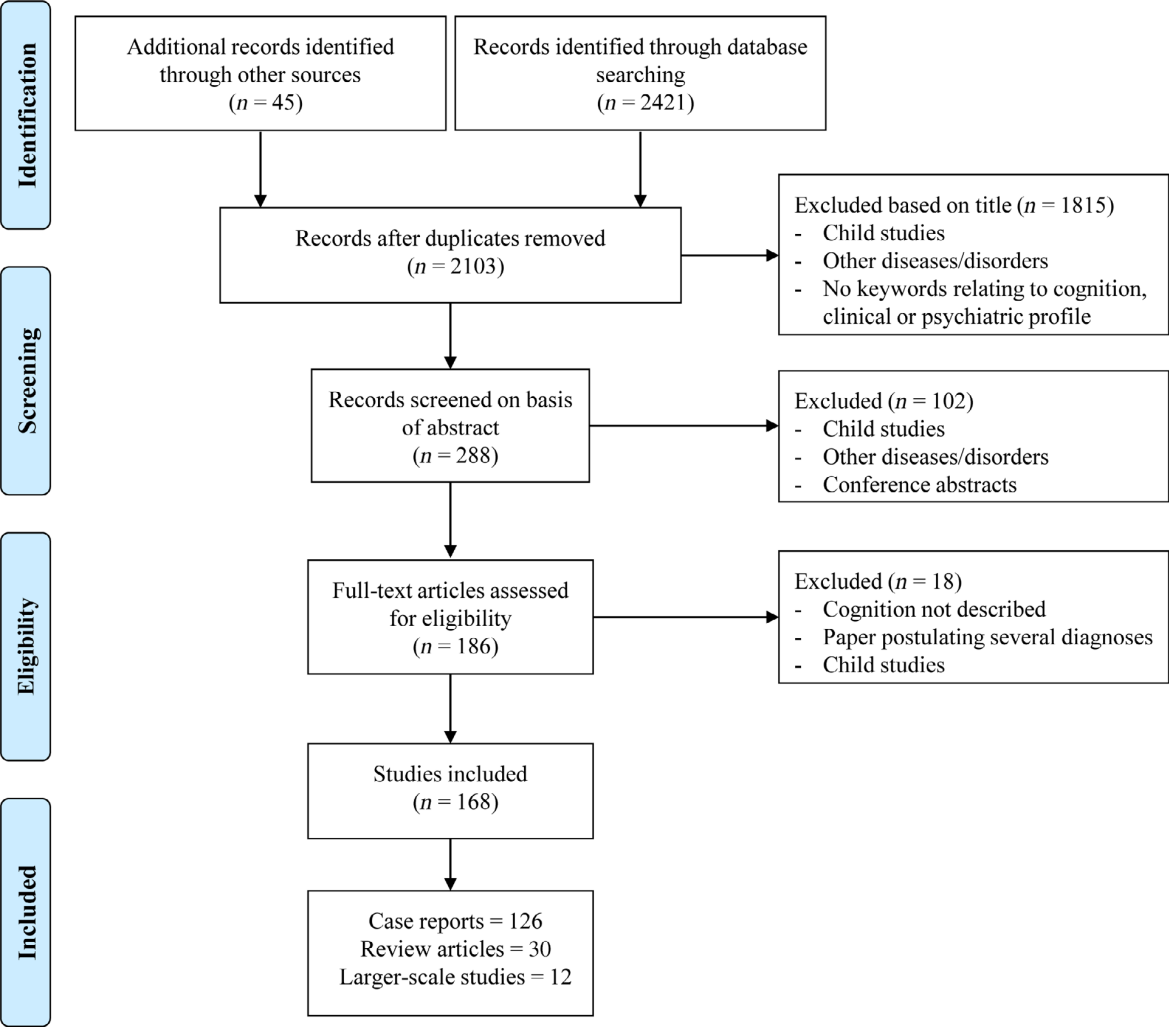
Process implemented to identify papers for this systematic review.

We independently searched five databases (PubMed, Medline, Psycinfo, Embase and Web of Science) for articles published up to and including 10 June 2019. Search terms related to mitochondrial disease and to cognitive functioning were chosen, based on the individual thesaurus used in each database (full search terms are provided in Tables S1–S5). Papers were further identified through manual search strategies and using 360‐degree citation methods to ensure that the breadth of the literature was comprehensively screened (Fig. 1).

### Review criteria

Inclusion criteria were English language, human studies of adult patients (≥16 years) with primary mitochondrial disease. Child studies were excluded unless part of a family study. No robust animal models yet exist for the common forms of mitochondrial disease associated with cognitive impairment; thus, animal research was excluded. Papers were considered if they referred to cognitive functioning in the title or abstract, or to discussion of clinical findings1Definitions of cognitive terms are as follows.
*Cognitive impairment:* difficulties in one or more domain of cognitive functioning, e.g. knowledge, attention, memory or reasoning.
*Developmental delay:* delays in reaching developmental milestones compared with expected levels for a child's age.
*Intellectual decline/cognitive decline:* decline in cognitive abilities from previous levels.
*Dementia:* decline in memory or other thinking skills severe enough to reduce a person's ability to perform everyday activities.
*Full‐scale IQ (FSIQ):* general cognitive abilities.
*Performance IQ (PIQ):* non‐verbal reasoning skills.
*Verbal IQ (VIQ):* verbal knowledge and reasoning skills.
*Memory:* ability to learn, remember and recall verbal information, e.g. remembering a shopping list or a conversation with a friend, family member or healthcare professional.
*Processing speed:* how quickly a person can take information in and use it.
*Executive function:* higher order control processes that guide behaviour, help you to plan what to do, monitor whether a plan is working, change plans if the first is not working or stop yourself from doing something that you should not.
*Visuospatial/visuoconstruction:* use of visual information to understand and interact with the environment, e.g. finding your way around a car park or putting together flat‐pack furniture.. Exclusion criteria were: other diseases/disorders commonly associated with mitochondrial dysfunction (e.g. Creutzfeldt–Jakob disease, Friedreich's ataxia, Parkinson's disease, Huntington's disease), diseases of memory without mitochondrial disease (e.g. Alzheimer's disease, dementia), psychiatric problems without concurrent cognitive difficulties, unpublished data, conference presentations and abstracts where the full paper was not published.

### Data synthesis

Due to substantial methodological and clinical heterogeneity in systematic research studies identified by this literature search (Table 1), we used a narrative approach to synthesize the findings of the included studies, as meta‐analysis was deemed inappropriate 12.

**Table 1:** Summary of systematic investigations of cognition in patients with mitochondrial disease

	Author	Sample	Follow‐up?	Cognitive assessments	Cognitive domain assessed	Definition of impairment	Baseline imaging
*m.3243A&gt;G* point mutations	Majamaa‐Voltti *et al*. 13	33 Average muscle heteroplasmy: 71% SD: 12% Age‐, sex‐ and education‐matched controls	3‐year follow‐up study	WAIS‐R WMS‐I Benton Visual Retention test TMT A and B Letter Fluency Clock and Three‐Dimensional Cube Copying task Finger Tapping test Reaction Time test (choice reaction)	Verbal and visual intelligence Verbal memory Visual memory Executive function Executive function Visual perception and construction Motor performance Motor performance	Five or more of the seven domains impaired	Brain atrophy: 45% Mild white matter changes: 26% Stroke lesions: 9% Basal ganglia calcifications: 12% EEG was normal in 67%, mildly abnormal in 21% and moderately abnormal in 12% of cases Follow‐up: MRI: 20 participants No changes in 16 participants EEG: 13 participants Significantly lower mean parietal and occipital EEG alpha waves
	Kaufmann *et al*. 14	85 Fully symptomatic = 31 Asymptomatic/partially symptomatic = 54	Single time point	Specific assessments not specified	N/A	Age‐adjusted global cognition score	N/A
	Kraya *et al*. 15	10 Healthy, age‐, gender‐ and education‐matched controls	MRI and cognition Single time point	Mehrfachwahl Wortschatz Intelligenztest B Rey Osterrieth Complex Figure test Regensburg Word Fluency Test Zoo Map task from the Behavioural Assessment of the Dysexecutive Syndrome Auditory Verbal Learning Test (AVLT) Digit span backwards from the WMS‐R TMT A/B Hospital Anxiety and Depression Scale	Pre‐morbid (crystalline) intelligence Visuoperception, visuoconstruction, secondary visual memory Executive function Executive function Verbal working memory Verbal working memory Selective attention Anxiety and depression		Most pronounced lesions found in temporo‐occipital, occipital and parietal regions No lesions in frontal regions Loss of grey and white matter: 90% (normal: 10%, slight abnormality: 60%, moderate abnormality: 30%)
MIDD	Fromont *et al*. 16	11 *m.3243A&gt;G* = 9 *m.14709T&gt;G* = 2 9 age‐matched, type 1 diabetic controls	Single time point	Mini‐Mental State Examination Hamilton test Free and Cued selective Reminding test Rey's Figure Visual memory subtest of the WMS‐R Digit Span subtest of the WAIS‐R TMT A/B Raven's Progressive Matrices PM‐38 Verbal Fluency Frontal Assessment Battery DO80, a standardized French picture‐naming test Rey's Figure	Global cognitive function Depression Episodic memory Immediate and delayed recall Visual memory Executive function Executive function Executive function Executive function Executive function Language Visual, spatial and constructive abilities	Z‐score below −1.65SD, standard score below six or percentile below 5%	Vermis atrophy: 70% Severe cerebellar atrophy extending into cerebellar hemispheres: 18% Focal white matter lesions: 50% Basal ganglia calcifications: 9%
Large‐scale single deletion	Turconi *et al*. 7	16 CPEO = 9 CPEO+ = 3 KSS = 3 MERRF = 1 Point mutation (unspecified) = 1 Multiple deletions = 2 Large‐scale single deletion = 13	Single time point	WAIS‐R Digit Span subtest of the WAIS‐R Memory Assessment Scale Judgment of Line Orientation Rey Figure (A and B) Copy test	VIQ, PIQ and FSIQ Verbal short‐term memory Global memory, short‐term memory, verbal memory and visual memory Visuoperceptual organization and drawing abilities Perceptual motor skills	FSIQ<70 (2SD from the norm)	MRI normal: 81% Diffuse aspecific abnormalities: 13% (2 patients with KSS)11 patients with single‐photon emission computed tomography results: At least one significant asymmetry: 100% Bilateral involvement: 45% CPEO/CPEO+: temporal cortex: 7/8 patients, mesial regions: 5/7 without prevalent laterality, basal ganglia: 4/8, thalamus (3/8) temporal lobes, mesial parieto‐occipital: 3/8 KSS: widespread cortical and subcortical hypoperfusion: 1/2, left mesial temporal cortex involvement: 1/2 MERRF: right thalamus involvement: 1/1
	Bosbach *et al*. 17	22 CPEO = 16 KSS = 6 Large‐scale single deletions = 15 *m.3243A&gt;G* = 2 Unknown = 5 Healthy age‐, gender‐ and education‐matched controls	Single time point		Language and visuoconstruction Language/aphasia screening Visual perception Visuoconstruction and visual memory Visuomotor coordination Perceptual speed/visual scanning Sustained attention and visual scanning Vigilance Abstraction/flexibility Abstraction/flexibility Verbal memory Health‐related quality of life	Score below the 10th percentile	N/A
Multiple deletions	Gramstad *et al*. 18	8 with MSCAE (one of two POLG1 mutations: *1399G&gt;A* or *2243G&gt;C*)	3‐year follow‐up of one patient	WAIS, Norwegian translations with US norms WMS‐R, Norwegian version Halstead–Reitan Battery, using Norwegian cross‐validation data	VIQ, PIQ and FSIQ. General Memory Index, Verbal Memory Index, Visual Memory Index Memory Brain and nervous system functioning	Not specified	N/A
Multiple genotypes	Lang *et al*. 19	15 PEO = 8 MELAS = 4 KSS = 3	Single time point	Neuropsychological Deficit Test Screening Battery	Memory, orientation, non‐verbal intelligence, drawing, arithmetic, word list generation, trail making and digit span	Not specified	N/A
	Montirosso *et al*. 20	11 with no known cognitive impairment CPEO = 2 CPEO+ = 6 Ptosis and myopathy = 3	Single time point	WAIS Auditory oddball paradigm of pure tones or phonetic stimuli, with EEG recorded at three midline sites (Fz, Cz, Pz)	Global cognitive function to exclude dementia Performance, reaction time and brain functioning	N/A	No increase in latency of event‐related potential N1, P2 or P3 components compared with controls Significant delay in latency of N2 component MRS venous lactate elevated in symptomatic and oligosymptomatic 3243A>G carriers compared with controls
		Large‐scale deletion = 5 Multiple deletions = 4 No diagnosis = 2 14 age‐matched controls					
	Kaufmann *et al*. 21	*m.3243A&gt;G* = 91 individuals from 34 families *m.8344A&gt;G*=15 individuals from two families	Single time point Categorized as either asymptomatic, partially (oligo)symptomatic or symptomatic	Specific assessments not specified		N/A	MRS ventricular lactate rose significantly from asymptomatic through to symptomatic 3243A>G carriers
	Inczedy‐Farkas *et al*. 22	19 *m.3243A&gt;G *= 4 *m.8344A&gt;G *= 4 *m.8993T&gt;C *= 2 Multiple mitochondrial DNA deletions = 3 Other (including multiple mutations) = 6 13 demographically similar, healthy controls	Single time point	WAIS Rey Auditory Verbal Learning Test Stroop Color‐Word Test TMT A/B Verbal Fluency	Intellectual abilities, not otherwise specified Verbal encoding, short‐ and long‐term recall and proactive and retroactive interference Verbal processing speed and susceptibility to interference Psychomotor speed and visual attention Phonemic and semantic fluency	Z‐score produced from control raw data	N/A
	Moore *et al*. 23	49 At baseline: *m.3243A&gt;G *= 36 *m.8344A&gt;G *= 13 32 age‐ and pre‐morbid cognitive ability‐matched controls	18‐month follow‐up	WAIS‐IV Delis‐Kaplan executive function system WMS‐IV Birt Memory and Information Processing Speed Battery	FSIQ, verbal comprehension, perceptual reasoning, working memory, processing speed Verbal and non‐verbal executive functions; tapping working memory, self‐monitoring, inhibition, task switching, cognitive flexibility, planning, rule learning and inhibition Verbal immediate, delayed and recognition memory for narrative and word pairs Motor speed	Z‐scores >1, 2 and 3 SD from the normative mean	N/A

CPEO, chronic progressive external ophthalmoplegia; EEG, electroencephalographic; FSIQ, full‐scale IQ; Fz, Pz, Cz, location of scalp electrodes defined using the international 10‐20 system for EEG; IQ, intelligence quotient; KSS, Kearns‐Sayre syndrome; MELAS, mitochondrial encephalopathy, lactic acidosis and stroke‐like episodes; MERRF, myoclonic epilepsy with ragged red fibers; MIDD, maternally‐inherited diabetes and deafness; MRI, magnetic resonance imaging; MRS, magnetic resonance spectroscopy; MSCAE, mitochondrial spinocerebellar ataxia and epilepsy; N/A, not applicable; PEO, progressive external ophthalmoplegia; PIQ, performance IQ; TMT, Trail‐Making Test; VIQ, verbal IQ; WAIS, Wechsler Adult Intelligence Scale; WAIS‐R, Wechsler Adult Intelligence Scale‐Revised; WMS‐I, Wechsler Memory Scale‐I; WMS‐IV, Wechsler Memory Scale‐IV; WAIS‐IV, Wechsler Adult Intelligence Scale‐IV; WMS‐R, Wechsler Memory Scale‐Revised.

## Results

The literature search yielded 2421 articles, of which 168 met inclusion criteria. Articles were subdivided into case literature (75% of published articles), reviews of the medical literature (18%) and larger scale, systematic investigations of cognition in mitochondrial disease (7%; Fig. 1), categorized by mutation/phenotype.

### Case literature

Many case reports have been published that give a clinical profile of the patient(s) in question, including a statement about cognition (Table 2), without reference to the neuropsychological assessments and/or results obtained (see Table S6 for broader impairments associated with cognitive difficulty and the reference list for both tables). Cognition was reported in 114 patients; 34% showed cognitive impairment, 24% cognitive decline and 15% dementia. Cognitive domains with high reports of impairment were memory (32%) and language (20%). However, to highlight variability in reporting, only 2% identified dementia and memory difficulties, 14% cognitive impairment and memory difficulties, and 7% cognitive decline and memory difficulties (which could constitute a diagnosis of dementia, depending on the extent of impairment).

**Table 2:** Case reports detailing cognitive difficulties

Author	Patient information				Cognitive difficulties												Other (Table S6)
	*n*	Age (years)	Sex	Genotype/ phenotype	No evidence of CI	Global impairment				Cognitive domains							
						Developmental delay	Dementia	Cognitive impairment	Cognitive decline	Comprehension^+^	Language^++^	Arithmetic	Memory	Processing speed	Executive function	Visuospatial	
Morgan‐Hughes *et al*.	1	46	M	NK											+		+
	1	48	F								+		+			+	+
Suzuki *et al*.	1	31	M	NK			+			+	+	+	+			+	+
Finsterer *et al*.	1	60	F	NK									+				+
Lewandowska *et al*.	2^a^	36	F	NK			+										
		3	F			+		+									
Gopal & Anand	1	20	F	NK				+									
Holliday *et al*.	3	28	M	NK			+										
		17	M						+								
		27	F						+				+				+
Majamaa *et al*.	4	27	M	3243A>G	+												
		28	M	3243A>G					+								
		37	M	12308A>G					+								
		32	M	NK					+								
Penn *et al*.	3^a^	33–70		3243A>G MELAS			+	+									
Onishi *et al*.	2	39	M	3243A>G MELAS			+										
		17	F				+										
Kishimoto *et al*.	1	43	M	3243A>G MELAS				+									
Gilchrist *et al*.	1	46	F	3243A>G MELAS						+							+
Di Trapani *et al*.	1	27	M	3243A>G MELAS				+									
Huang *et al*.	1	28	M	3243A>G MELAS				+									
Kimata *et al*.	1	60	M	3243A>G MELAS					+		+		+				+
Sharfstein *et al*.	1	55	F	3243A>G MELAS					+	+	+				+		+
Silvestri *et al*.	2	18	F	3243A>G MELAS			+		+								
		52	F		+												
Feddersen *et al*.	2	43	M	3243A>G MELAS													+
		57	F								+						+
Conway *et al*.	1	29	F	3243A>G MELAS				+									+
Emmanuele *et al*.	4	37	F	3243A>G MELAS				+					+				
		39	M					+					+				+
		35	F					+					+				+
		38	F					+					+				
Benninger *et al*.	1	63	F	3243A>G MELAS				+									+
Collorone *et al*.	1	47	M	3243A>G MELAS							+						+
Prasad *et al*.	12^a^			3243A>G MELAS		+	+										+
Sparaco *et al*.	2	53	M	3243A>G MELAS					+		+						+
						+			+		+						
Dubeau *et al*.	1	31	F	3243A>G MELAS		+		+									
Fang, Zheng & Zhang	1	63	F	3243A>G MELAS							+						+
Smith *et al*.	1	61	F	3243A>G MELAS							+						
Isozumi *et al*.	1	50	F	MELAS			+	+			+						
Tsuchiya *et al*.	1	20	F	MELAS			+										
Aharoni *et al*.	4^a^	52	F	MELAS					+		+						+
	−>3	<18				+											
Kaufman *et al*.	1	39	M	MELAS					+								+
Köller *et al*.	1	37	M	MELAS									+				+
Apostolova *et al*.	1	58	F	MELAS									+		+	+	+
Ducreux *et al*.	1	23	M	MELAS		+			+								
Chu *et al*.	1	30	M	MELAS					+				+		+	+	
De Luca *et al*.	1	29	F	MELAS							+						
Marques‐Matos *et al*.	1	50	M	MELAS											+		
Seyama *et al*.	1	29	M	MELAS			+										
Rusanen *et al*.	1	38	M	3243A>G				+									
Dai *et al*.	1	37	M	3243A>G				+									
Pröbstel *et al*.	1	60	M	3243A>G				+									
Dickerson *et al*.	1	61	F	13513G>A MELAS			+			+	+				+		+
Lindberg *et al*.	1	44	F	7512T>C MELAS				+	+				+	+			
Connolly *et al*.	27^a^	4–47		3260A>G MELAS							+						+
Wang *et al*.	3	22	F	13513G>A MELAS/LS							+						
		16	M			+											+
		11	F			+				+		+	+				+
van den Ouweland *et al*.	11^a^	19–58		3243A>G MIDD	+												
Chen *et al*.	1	48	F	MIDD	+												
Lien *et al*.	6^a^	8–74		MIDD	+												
Kobayashi *et al*.	2^a^	41–67		3243A>G MIDD	+												
Herrero‐Martin *et al*.	1	50	F	5521G>A MELAS/ MERRF							+		+				+
Huang *et al*.	8	19–50		MELAS/ MERRF				+									
Han *et al*.	2^a^	38	F	8344A>G				+					+				
		42	F										+				
Larsson *et al*.	2	21	M	MERRF													+
		20	M				+										
Teive *et al*.	1	52	M	MERRF				+	+				+				
Taylor *et al*.	1	29	F	MERRF			+		+				+		+	+	
Mancuso *et al*.	1	42	F	611G>A MERRF				+					+				
Young *et al*.	1	57	F	586G>A				+			+		+	+	+		+
Morten *et al*.	1	31	F	3252A>G		+	+										
Amemiya *et al*.	1	29	M+	3256C>T			+										+
Jaksch *et al*.	1	33	M	3274A>G		+							+				+
Silvestri *et al*.	1	36	F	5540G>A			+										
Nelson *et al*.	1	45	M	5549G>A			+		+								+
Djordjevic *et al*.	1	24	F	5577C>T					+		+						+
Scuderi *et al*.	1	30	F	5814A>G				+			+						+
Bidooki *et al*.	1	36	F	7480A>G				+	+				+				
Koubeissi *et al*.	1	42	F	8296A>G	+												
Houshmand *et al*.	1	48	F	8328G>A				+						+	+		
Sano *et al*.	1	31	M	8356T>C				+	+								
Hanna *et al*.	1	36	F	9952G>A													+
Mezuki *et al*.	1	55	M	10158T>C ND3				+									
Taylor *et al*.	1	42	M	10191T>C in ND3					+								
Deschauer *et al*.	1	67	M	11777C>A in ND4					+								+
Coku *et al*.	1	35	F	12276G>A							+						+
Slawek *et al*.	1	21	M	13042G>A									+				
Schinwelski *et al*.	1	26	M	13042G>A				+									
Sikorska *et al*.	17^a^			4052T>C & 9035T>C		+											
Corona *et al*.	4^a^	27–64		4284G>A	+				+								+
Santorelli *et al*.	12^a^	16–70		8363G>A				+	+								
	7^a^	24–44						+									+
Virgilio *et al*.	7^a^	29–65		8363G>A		+		+									+
Tsao *et al*.	14^a^	6–58		8993T>G	+			+	+		+		+				+
Arenas *et al*.	5^a^	27–62		8296A>G & 8363G>A													+
Wei & Wang	1	26	M	m.9176T>C	+												
Shoffner *et al*.	1	28	M	tRNA deletion 3271–3273													+
Debray *et al*.	1	18	F	7402delC					+								
De Coo *et al*.	1	20	M	4‐bp deletion at 14787							+				+		+
Nishihara *et al*.	1	60	F	2‐bp deletion in C12orf65		+		+									
Puoti *et al*.	1	48	F	KSS					+								
Van Goethem *et al*.	1	18	M	*POLG*					+								
Mancuso *et al*.	1	48	M	*POLG*					+								
Luoma *et al*.	1	44	F	*POLG*									+				+
Deschauer *et al*.	1	28	M	*POLG1*									+				+
Van Hove *et al*.	1	71	F	*POLG*									+	+			+
Martikainen *et al*.	1	64	F	c.2993C>T & c.3550G>C in *POLG1*			+	+									+
Synofzik *et al*.	2^a^	32	F	W748S in *POLG*				+									
		40	F					+	+								
Hakonen *et al*.	2	31	F	W748S & E1143G in *POLG*				+	+		+		+			+	+
		57	M					+					+				+
Hansen *et al*.	1	23	F	p.A467T & p.W748S in *POLG1*							+						
Hudson *et al*.	6^a^	41–74		*POLG1*			+										
Melberg *et al*.	2^a^	60–61		*POLG1*					+				+			+	
Bee *et al*.	2	48	M	c.673C>T				+					+		+	+	+
		48	M								+		+				+
Komulainen *et al*.	3^a^	78–86		c.2447G>A (p.R722H) in *POLG1*			+		+				+				+
	2^a^	17–22						+									+
Rantamäki *et al*.	41^a^	19–84		W748S & A467T in *POLG*			+										+
Echaniz‐Laguna *et al*.	2^a^	81–82		R374W in *PEO1*			+		+								
	3^a^	42–64			+												
Gebus *et al*.	3^a^	NK	M	W748S & R627Q in *POLG*	+												
		58	M		+												
		NK	F					+									
Bianco *et al*.	1	24	M	m.3460G>A				+									
Morimoto *et al*.	1	37	F	LHON				+					+				+
Hirano *et al*.	1	40	M	MNGIE				+					+				
Carod‐Artal *et al*.	1	35	M	MNGIE				+					+				
Martí *et al*.	1	61	F	MNGIE									+				+
Bariş *et al*.	1	18	F	MNGIE													+
Spiegel *et al*.	4^a^	7–20		MNGIE													+
Schuepbach *et al*.	3^a^	20–22		MNGIE	+												
Blondon *et al*.	3			MNGIE	+												
Massa *et al*.	1	67	F	MNGIE									+			+	

a Family study (+, deficit in ≥1 family member).

+, comprehension including ability to follow commands; ++, language, including reading and writing; CI, cognitive impairment; F, female; KSS, Kearns‐Sayre syndrome; LHON, Leber's hereditary optic neuropathy; LS, Leigh syndrome; M, male; MELAS, mitochondrial encephalopathy, lactic acidosis and stroke‐like episodes; MERRF, myoclonic epilepsy with ragged red fibers; MIDD, maternally‐inherited diabetes and deafness; MNGIE, mitochondrial neurogastrointestinal encephalomyopathy; NK, not known; PEO, progressive external ophthalmoplegia.

Comprehensive assessment of 14 patients with MELAS revealed higher VIQ than PIQ, and intellectual decline and dementia 24, 25, as well as visuospatial difficulties 25, 26, attention difficulties, verbal fluency, set‐shifting ability, planning and problem solving (suggestive of executive dysfunction) 26, 27, auditory agnosia (inability to process environmental sounds and speech, in the absence of aphasia) 28, 29, 30, 31, 32 and understanding of sentences 26. Magnetic resonance imaging (MRI) of the brain generally revealed generalized atrophy 24, 25, 26, 28 although electroencephalographic results from one study were suggestive of focal lesions 27. Cognitive decline was commonly reported in patients with point mutations 33, 34, 35. Wide‐ranging difficulties were described, with all reports identifying deficits in visuospatial functioning, memory and attention 33, 34, 35, 36, 37, but with variation in impairment in language 33, 34, 35, processing speed 36, 37, verbal fluency, planning, and initiative and set‐shifting (areas of executive functioning) 35, 36, 37. Relative strengths varied across patients, with some showing better verbal 37 and some showing better visual 33, 34 abilities.

### Reviews of the medical literature

A number of studies have reviewed different domains of cognitive functioning in patients with mitochondrial disease, often reporting variable frequencies of individuals with dementia, cognitive impairment and cognitive decline (Table 3).

**Table 3:** Prevalence of cognitive difficulties reported by review articles^a^

Author	Phenotype	*n*	Cognitive difficulties
Goto *et al*.	MELAS	40	65% showed cognitive impairment
Hirano *et al*.	MELAS	69	Dementia reported in 90% of cases Learning disability in 60% of cases
Damian *et al*.	MELAS	21	Dementia/cognitive impairment in 5/21 cases
Chinnery *et al*.	MELAS	111	27% of cases presented with dementia
Majamaa *et al*.	MELAS	11	4/11 patients showed cognitive decline
Suzuki *et al*.	m.3243A>G	113	7.1% showed cognitive impairment 3.6% presented with dementia
Murakami *et al*.	m.3243A>G	14	Current cognitive decline in 6/8 patients with prior diagnosis of diabetes (75%) and 0/6 without prior diagnosis of diabetes
Chae *et al*.	MELAS	18	Learning disability reported in 50% of cases
Sproule & Kaufmann	MELAS	45	Memory problems reported in 71% of cases
Lorenzoni *et al*.	MELAS	10	Developmental delay reported in 1/10 patients Dementia present in 5/10 patients
Ma *et al*.	MELAS	47	33/47 probands (70.2%) showed cognitive impairment
Hammans *et al*.	MERRF	18	Dementia in 22% of cases
Ozawa *et al*.	MERRF	10	Cognitive deterioration in 50% of cases
Chinnery *et al*.	MERRF	55	25% of cases presented with dementia
Sinha *et al*.	MERRF	10	Cognitive decline in 7/10 patients
Lorenzoni *et al*.	MERRF	6	6/6 had normal early development. 2/6 presented with of dementia
Mancuso *et al*.	MERRF	34	2/34 presented with cognitive involvement (6%)
Pavlakis *et al*.	MELAS/MERRF/KSS	97	9/11 MELAS, 11/16 MERRF, 34/70 KSS presented with dementia
Khambatta *et al*.	KSS	35	11/35 presented with cognitive decline
Wray *et al*.	KSS	8	4/8 presented with cognitive impairment
Van Goethem *et al*.	Multiple deletions	8	Unspecified number with mild cognitive impairment
Winterthun *et al*.	Multiple deletions	6	5/6 patients exhibited cognitive dysfunction
Hakonen *et al*.	Multiple deletions	6	Mild to moderate cognitive impairment in 74% of cases
Horvath *et al*.	Multiple deletions	19	Early‐onset dementia in 11% of cases
Tzoulis *et al*.	Multiple deletions	26	Suspected mild cognitive impairment in 8 patients, confirmed mild cognitive impairment in 4 patients
Wong *et al*.	Multiple deletions	61	Developmental delay or dementia reported in 66% of patients
Van Hove *et al*.	Multiple deletions	56	Memory loss reported in 5% of patients
Naïmi *et al*.	Multiple deletions and depletions	15	1/15 presented cognitive impairment
Jaksch *et al*.	Multiple point mutations	16	8/16 patients from 7 families presented with cognitive impairment

a References available in Appendix S1. KSS, Kearns‐Sayre syndrome; MELAS, mitochondrial encephalopathy, lactic acidosis and stroke‐like episodes; MERFF, myoclonic epilepsy with ragged red fibers.

Kartsounis and colleagues 38 reviewed the medical records of 72 patients with undefined mitochondrial disease from 1969 to 1989. General or focal cognitive deficits were observed in 61% of 36 patients who underwent neuropsychological assessment. Moderate to severe general intellectual decline was detected in 36%, defined by a discrepancy between estimated optimal intelligence quotient (IQ) and performance on general intelligence tests of ≥15 IQ points (15–30, moderate; 30+, severe). A total of 16 patients demonstrated memory deficits, 15 demonstrated perception deficits and 12 demonstrated language deficits. Only 28% displayed normal higher cerebral functioning. However, patients were assessed as part of clinical care and it is not known what the cognitive ability of untested individuals was, or whether they might have exhibited subtle cognitive deficits that were unlikely to be detected by initial clinical impression. This study provided neither phenotype/genotype reflecting the pre‐genetic era, with only four reporting genotype (Table 3) 39, 40, 41, 42.

### Larger‐scale, systematic investigations of cognition

A total of 12 studies conducted systematic investigations of cognition in mitochondrial disease (Table 1).

Three studies investigated cognition in patients with m.3243A>G. At initial assessment, Majamaa‐Voltti *et al*. 13 identified 16 patients as cognitively impaired (50%). In the whole sample, executive dysfunction was commonly observed, with 14 patients (14/32) failing to complete the task. Cognitive progression was rare, although data were not provided to corroborate this.

Kaufmann *et al*. 14 demonstrated that their fully symptomatic patient group (*n* = 31) not only performed worse than the asymptomatic/partially symptomatic group but also showed progressively worse cognition over time, whereas the asymptomatic/partially symptomatic group remained stable. However, specific assessments and results of cognitive domains were not reported. Furthermore, high dropout rates were evident between years 1 and 4, limiting the generalizability of results over time.

Kraya *et al*. 15 found that patients performed significantly worse than controls on measures of visuoconstruction, Trail‐Making Test (TMT) visual and divided attention, set‐switching/inhibition and verbal fluency, but not on other measures of executive function, working/visual memory. Higher lesion load correlated with worse attention, visuoconstruction, list‐learning ability and verbal fluency. Increasing symptom severity (measured by Newcastle Mitochondrial Disease Adult Scale 43 correlated with worsening attention, planning, verbal fluency and list learning.

One study investigating cognition in patients in association with maternally‐inherited diabetes and deafness (MIDD) (m.3243A>G:9, m.14709T>G:2) 16 found executive functioning and visual memory deficits. The authors suggested that visual memory impairment may be caused by executive dysfunction, particularly as verbal memory was intact using a task that minimized executive function load. Although cognitive deficits were found in patients with MIDD, no cognitive deficit was severe enough to fit dementia criteria, as demonstrated by Mini‐Mental State Examination scores within the normal range. When compared with type 1 diabetics, patients with MIDD showed worse attention, working memory and abstract reasoning.

Two studies explored cognition in patients with large‐scale single mitochondrial DNA deletions. Turconi *et al*. 7 found no impairment in FSIQ; however, PIQ was lower than VIQ. Specific deficits were also found in short‐term memory and visuospatial abilities. Bosbach *et al*. 17 observed no general intellectual decline or dementia compared with controls (Table 1). However, specific deficits were found in executive function, attention and visual construction. Examination of quality of life demonstrated differences from the norm on a number of measures; however, subjective wellbeing and limitations in social activities did not differ significantly.

Only one study has investigated cognition in patients with POLG‐related mitochondrial disease (N:8; mitochondrial spinocerebellar ataxia and epilepsy phenotype). Gramstad *et al*. 18 found a mean FSIQ of 77.4 (moderate impairment) and all patients showed significantly better VIQ than PIQ. This led the authors to suggest reduced cognitive functioning, although five patients scored within the normal range. Retesting one patient 3 years later showed declines in FSIQ, VIQ and PIQ. A trend of lower IQ with increasing age at assessment and duration of epilepsy was noted, suggesting a profile of progressive cognitive decline. Unfortunately, no statistical analyses were reported to corroborate these claims.

Five systematic investigations of cognition have included patients with variable genotypes. The assessments of Lang *et al*. 19 showed that patients with KSS performed best and those with MELAS performed worst (PEO, 8; MELAS, 4; KSS, 3). Four out of 15 patients performed within the normal range (KSS, 2; PEO, 2) and seven patients were impaired on more than half of all subtests (PEO, 3; MELAS, 4). Patients showed the highest number of deficits on TMT, suggesting difficulties in tasks tapping executive functions, although patient scores may have been affected on the TMT by ophthalmoplegia or motor speed difficulties (not considered by the authors). Over half of all patients demonstrated memory impairment. Five patients (MELAS, 3; PEO, 2) were diagnosed with dementia. Although patients with MELAS were more severely affected than patients with kearns‐sayre syndrome KSS or progressive external ophthalmoplegia PEO, there appeared to be no readily identifiable pattern of deficits.

Montirosso *et al*. 20 demonstrated performance and reaction time for patients (Table 1) and controls on both tasks. However, electroencephalographic activity showed a delayed N2 component, reflecting early slowing of information processing, and reduced P3 amplitude, highlighting dysfunction in post‐perceptual resetting and updating of information. These results indicated processing‐speed problems at a neuronal level, in the absence of cognitive impairment.

Kaufmann *et al*. 21 completed comprehensive neuropsychological testing on 91 individuals from 34 m.3243A>G families and 15 individuals from two m.8344A>G families. They identified progressively worse neuropsychological performance in asymptomatic, oligosymptomatic and symptomatic groups, which correlated with estimates of brain ventricular lactate. Cerebral lactate correlated not only with global cognitive functioning, but also with tests specifically measuring frontal brain regions, which are rarely affected by stroke‐like episodes. Furthermore, m.3243A>G probands showed lower neuropsychological test scores than m.8344A>G probands, suggesting genotypic variance in cognitive profiling. Although this study assessed a large group of individuals, information about the specific neuropsychological tests and their results was limited.

Inczedy‐Farkas *et al*. 22 found average FSIQ within normal limits, and lower PIQ than VIQ scores. Pronounced PIQ deficits were observed in processing speed and visuospatial functioning. Despite average FSIQ scores, the authors reported a general pattern of moderate to severe cognitive impairment. Memory performance was variable, with recall being differentially more impaired than encoding/learning and retroactive (but not proactive) interference. Although data from 13 control participants were collected, parametric statistical comparisons were not made with patients due to genetic heterogeneity. Comparisons of effect sizes were used, however, criteria of magnitude were not defined.

Our group 23 showed that patients had mild to moderate pre‐morbid cognitive impairment, but substantial impairment in FSIQ, as well as distinct domains of verbal comprehension, perceptual reasoning, working memory, processing speed and memory. It was not clear whether the executive dysfunction reported in this sample was caused by frontal difficulties, or simply slower decision‐making. These results were corroborated by significant differences from controls, except for memory, where performance was similar. This suggests that, in this sample, memory was intact when pre‐morbid cognition was controlled for. Cognitive decline appeared slow and was unlikely in the short‐term when other disease‐specific factors remained stable.

## Discussion

This is the first review to report a systematic search of the literature describing mitochondrial disease and cognition. Small sample sizes, genotypic variability and the breadth and depth of assessments undertaken introduced significant heterogeneity across studies precluding a meta‐analysis, as any interpretation would not be meaningful and could introduce significant bias. As such, our results provide a narrative synthesis of the literature.

Examination of the case literature commonly revealed dementia, cognitive impairment and cognitive decline, as well as other memory problems in patients with mitochondrial disease. Difficulties with language and attention were frequently reported, as well as personality and mood disorders, whereas executive dysfunction was rarely reported, although this was identified more frequently with detailed assessment. Reviews of medical reports provided broad diagnoses of impairment, and dementia, learning disability, cognitive impairment and cognitive decline were reported as common. It is difficult to know whether the identified impairments represent the common difficulties experienced by patients with mitochondrial disease, or a more extreme end of functioning of severely affected individuals who have warranted clinical evaluation and intervention.

Systematic studies that have reported the results of neuropsychological testing are much less indicative of general cognitive impairment and dementia than is suggested by the findings of case reports and review articles. These results instead provide strong support for a profile of focal cognitive deficits, rather than global difficulties, in areas such as visuospatial functioning, memory, attention, processing speed and executive functions. In terms of clinical care, detailed descriptions of focal cognitive difficulties are much more beneficial, as this information can feed into the development of management strategies. Further difficulties arise with understanding the cognitive challenges faced by patients with mitochondrial disease as a group because the literature does not currently apply a uniform classification system for the severity of cognitive deficit. There has also been substantial variability in the range of cognitive assessments used across systematic research studies identified by this review and little discussion about suitability of measures. Consensus on this matter is challenging due to the clinical heterogeneity found within this group of diseases; however, consideration must be given to the physical limitations of the sample in question and how these might affect performance. As an example, performance on standard versions of the TMT 44 might be affected by ocular movements across the test page, ataxia, muscular weakness and fatigue.

Although the evidence is not strong enough to substantiate distinct genotype‐specific cognitive profiles, research considering genotypic variation in cognitive functioning has postulated differences 19, 21. However, with the exception of Kaufmann and colleagues 21, most systematic investigations have remained small, and clinical profiles and/or genetic mutations have varied considerably, limiting the ability to generalize these findings to the wider population of patients with mitochondrial disease. Currently, understanding about the aetiology and progression of cognitive dysfunction is limited. Virgilio and colleagues 45 found significantly higher mutation load in patients with a severe clinical phenotype than mild/moderate and asymptomatic phenotypes, but they did not explore the link with cognitive functioning. More recently, our group 23 showed that disease severity (measured by Newcastle Mitochondrial Disease Adult Scale and urinary heteroplasmy), rather than genotype, is a stronger predictor of cognitive abilities. Research has also shown links between severity of other symptoms related to mitochondrial disease (e.g. cerebral lactate, nuclear factors) and general cognitive performance 21, 46, but specific cognitive domains have not been robustly investigated. As highlighted in Table 1, neuroimaging findings (where available) vary greatly across studies. The current literature does not clarify whether focal cognitive deficits and general cognitive impairment/dementia are part of a continuum of symptoms varying in severity, and how these relate to focal lesions and generalized atrophy in patients, nor how underlying mutation load accounts for these changes. Unfortunately, neuroimaging data are too scarce at present to determine the association between cognitive difficulties and imaging results. Another important consideration when interpreting cognitive dysfunction is additional disease factors that may exacerbate cognitive performance on any given day (e.g. fatigue, mental health difficulties). Unfortunately, no systematic research to date has addressed this.

Information about prognosis is also currently limited, as most research investigating disease‐specific factors related to cognitive impairment has taken measurements at a single time point. Samples in longitudinal studies, to date, remain small and require validation. Kaufmann *et al*. 14 found worse initial performance and greater progression of general cognitive difficulties over a 4‐year period in fully symptomatic vs. asymptomatic/partially symptomatic (relatives) patients with m.3243A>G mutations. Conversely, our group showed no change over 18 months when other disease‐specific factors remained stable, although decline between pre‐morbid and current cognition over the longer term was predicted by disease severity. Samples in longitudinal research remain small, and greater follow‐up duration is required to validate results. This is essential for understanding disease progression and trajectories of change, in order to apply this to clinical monitoring and subsequent health planning.

## Conclusions

From this systematic review, patients with mitochondrial disease face high rates of cognitive difficulties, in areas including (but not limited to) visuospatial functioning, memory, attention, processing speed and executive functions. However, there is a great deal of variation in the specific deficits reported, and the pattern and progression are not fully understood. In order to advance this field, larger samples of well‐characterized patients should be assessed using comprehensive, systematic measures tapping different domains of cognitive functioning, including real‐time functional imaging during assessments.

## Disclosure of conflicts of interest

The authors declare no financial or other conflicts of interest.

## Supporting information


**Table S1.** OVID Medline electronic search term strategy (1946 to 12 November 2018).
**Table S2.** PsycINFO electronic search term strategy (1806 to November Week 1 2018).
**Table S3.** Embase electronic search term strategy (1974 to 12 November 2018).
**Table S4.** PubMed electronic search term strategy (1970–2018).
**Table S5.** Web of Knowledge electronic search term strategy (1970–2018).

**Table S6.** Additional impairments found in patients with mitochondrial disease experience who also experience cognitive difficulties.

**Appendix S1.** References.
