# Supplementary material for: Systematic review of cognitive deficits in adult mitochondrial disease
Source: Eur J Neurol. 2019 Oct 22;27(1):3–17. doi: 10.1111/ene.14068 (PMC6916601; doi:10.1111/ene.14068)
Supplement: Supplementary file 1 — Table S1. OVID Medline electronic search term strategy (1946 to 12 November 2018). Table S2. PsycINFO electronic search term strategy (1806 to November Week 1 2018). Table S3. Embase electronic search term strategy (1974 to 12 November 2018). Table S4. PubMed electronic search term strategy (1970–2018). Table S5. Web of Knowledge electronic search term strategy (1970–2018). [file ENE-27-3-s001.docx]

Online Table 1. OVID Medline electronic search term strategy (1946 to June 07, 2019).

| **#** | **Search Terms** |
| --- | --- |
| 1 | Exp Mitochondrial Diseases |
| 2 | Exp Cognition OR Cognition Disorder |
| 3 | Executive Function |
| 4 | Exp Memory |
| 5 | Mental Processes |
| 6 | Reaction Time |
| 7 | Language |
| 8 | Dementia |
| 9 | Exp Psychological Tests |
| 10 | Exp Neuropsychological Tests |
| 11 | 2 or 3 or 4 or 5 or 6 or 7 or 8 or 9 or 10 |
| 12 | 1 and 11 |
| 13 | Limit 12 to (English language and Humans and ("young adult (19 to 24 years)" or "adult (19 to 44 years)" or "young adult and adult (19-24 and 19-44)" or "middle age (45 to 64 years)" or "middle aged (45 plus years)" or "all aged (65 and over)" or "aged (80 and over)")) |

Online Table 2. PsycINFO electronic search term strategy (1806 to June Week 1 2019).

| **#** | **Search Terms** |
| --- | --- |
| 1 | Mitochondria |
| 2 | Mitochondrial disease.mp |
| 3 | Mitochondrial encephalomyopathies.mp |
| 4 | Mitochondrial cytopathies.mp |
| 5 | Exp MELAS |
| 6 | MERRF.mp |
| 7 | Encephalopathies |
| 8 | 1 or 2 or 3 or 4 or 5 or 6 or 7 |
| 9 | Exp Cognition |
| 10 | Exp Memory OR Memory Disorders |
| 11 | Language OR Language Disorders |
| 12 | Exp Executive function |
| 13 | Exp Cognitive processing speed |
| 14 | Exp Reaction time |
| 15 | Dementia |
| 16 | Exp Cognitive ability |
| 17 | Exp Intelligence |
| 18 | Neuropsychological assessment |
| 19 | 9 or 10 or 11 or 12 or 13 or 14 or 15 or 16 or 17 or 18 |
| 20 | 8 and 19 |
| 21 | Limit 20 to (Human and English language and Adulthood <18+ years>) |

Online Table 3. Embase electronic search term strategy (1974 to 2019 June 07).

| **#** | **Search Terms** |
| --- | --- |
| 1 | Exp “Disorders of mitochondrial functions” |
| 2 | Exp Cognition |
| 3 | Exp Memory OR Exp Long term memory |
| 4 | Exp Language |
| 5 | Exp Executive function |
| 6 | Exp Attention |
| 7 | Reaction time |
| 8 | Dementia |
| 9 | Exp Cognitive defect |
| 10 | Exp Intellectual impairment |
| 11 | Exp Mental deterioration |
| 12 | Exp “Disorders of higher cerebral function” |
| 13 | 2 or 3 or 4 or 5 or 6 or 7 or 8 or 9 or 10 or 11 or 12 |
| 14 | 1 and 13 |
| 15 | Limit 14 to (Human and English language and (adult <18 to 64 years> or aged <65+ years>)) |

Online Table 4. PubMed electronic search term strategy (1970-2019).

| **#** | **Search Terms** |
| --- | --- |
| 1 | mitochondrial disease OR MELAS OR MERFF OR mitochondrial cytopath* |
| 2 | cogniti* OR (cognitive (decline OR deterioration)) OR Memory OR Executive function OR Language OR processing speed OR dementia |
| 3 | 1 and 2 |
| 4 | Limit 3 to (Humans; English; Adult: 19+ years; Adult: 19-44 years; Aged: 65+ years) |

Online Table 5. Web of Knowledge electronic search term strategy (1970-2019).

| **#** | **Search Terms** |
| --- | --- |
| 1 | Neuropsy* test mitochondri* disease |
| 2 | Neuropsy* function mitochondri* disease |
| 3 | Neuropsy* function mitochondri* cytopath* |
| 4 | Cogniti* function mitochondri* cytopath* |
| 5 | Cogniti* mitochondri* cytopath* |
| 6 | 1 or 2 or 3 or 4 or 5 |
| 7 | Limit 6 to (Language=English) |
