# Supplementary material for: Systematic review of cognitive deficits in adult mitochondrial disease
Source: Eur J Neurol. 2019 Oct 22;27(1):3–17. doi: 10.1111/ene.14068 (PMC6916601; doi:10.1111/ene.14068)
Supplement: Supplementary file 2 — Table S6. Additional impairments found in patients with mitochondrial disease experience who also experience cognitive difficulties. [file ENE-27-3-s002.docx]

Online Table 7. Additional impairments found in patients with mitochondrial disease experience who also experience cognitive difficulties.

| **Author** | **Patient Information** | | | | **Daily Functioning** | | | **Associated Difficulties** | |
| --- | --- | --- | --- | --- | --- | --- | --- | --- | --- |
|  | **N** | **Age** | **Sex** | **Genotype/ Phenotype** | **Activities of Daily Living** | **Confusion** | **Attention/ Orientation** | **Social Functioning** | **Personality/ Mood** |
|  | 1 | 46 | M | NK | + | + |  |  |  |
| Morgan-Hughes et al(1) | 1 | 48 | F |  |  | + | + |  |  |
| Suzuki et al.(2) | 1 | 31 | M | NK |  |  |  | + | + |
| Finsterer et al.(3) | 1 | 60 | F | NK |  | + |  |  | + |
| Lewandowska et al.(4) | 2* | 36 | F | NK |  |  |  |  |  |
|  |  | 3 | F |  |  |  |  |  |  |
| Gopal and Anand(5) | 1 | 20 | F | NK |  |  |  |  |  |
| Holliday et al.(6) | 3 | 28 | M | NK |  |  |  |  |  |
|  |  | 17 | M |  |  |  |  |  |  |
|  |  | 27 | F |  |  |  |  |  | + |
| Majamaa et al.(7) | 4 | 27 | M | 3243A>G |  |  |  |  |  |
|  |  | 28 | M | 3243A>G |  |  |  |  |  |
|  |  | 37 | M | 12308A>G |  |  |  |  |  |
|  |  | 32 | M | NK |  |  |  |  |  |
| Penn et al.(8) | 3* | 33-70 |  | 3243A>G MELAS |  |  |  |  |  |
| Onishi et al.(9) | 2 | 39 | M | 3243A>G MELAS |  |  |  |  |  |
|  |  | 17 | F |  |  |  |  |  |  |
| Kishimoto et al.(10) | 1 | 43 | M | 3243A>G MELAS |  |  |  |  |  |
| Gilchrist et al.(11) | 1 | 46 | F | 3243A>G MELAS |  |  |  |  | + |
| Di Trapani et al.(12) | 1 | 27 | M | 3243A>G MELAS |  |  |  |  |  |
| Huang et al.(13) | 1 | 28 | M | 3243A>G MELAS |  |  |  |  |  |
| Kimata et al.(14) | 1 | 60 | M | 3243A>G MELAS |  | + |  |  |  |
| Sharfstein et al.(15) | 1 | 55 | F | 3243A>G MELAS |  |  |  |  | + |
| Silvestri et al.(16) | 2 | 18 | F | 3243A>G MELAS |  |  |  |  |  |
|  |  | 52 | F |  |  |  |  |  |  |
| Feddersen et al.(17) | 2 | 43 | M | 3243A>G MELAS |  | + |  |  | + |
|  |  | 57 | F |  |  | + |  |  | + |
| Conway et al.(18) | 1 | 29 | F | 3243A>G MELAS |  | + |  |  |  |
| Emmanuele et al.(19) | 4 | 37 | F | 3243A>G MELAS |  |  |  |  |  |
|  |  | 39 | M |  |  |  | + |  | + |
|  |  | 35 | F |  |  |  | + |  | + |
|  |  | 38 | F |  |  |  |  |  |  |
| Benninger et al.(20) | 1 | 63 | F | 3243A>G MELAS |  | + |  |  |  |
| Collorone et al.(21) | 1 | 47 | M | 3243A>G MELAS |  | + |  |  | + |
| Prasad et al.(22) | 12* |  |  | 3243A>G MELAS |  |  |  |  | + |
| Sparaco et al.(23) | 2 | 53 | M | 3243A>G MELAS |  |  |  |  | + |
|  |  |  |  |  |  |  |  |  |  |
| Dubeau et al.(24) | 1 | 31 | F | 3243A>G MELAS |  |  |  |  |  |
| Fang, Zheng, and Zhang(25) | 1 | 63 | F | 3243A>G MELAS |  |  |  |  | + |
| Smith et al.(26) | 1 | 61 | F | 3243A>G MELAS |  |  |  |  |  |
| Isozumi et al.(27) | 1 | 50 | F | MELAS |  |  |  |  |  |
| Tsuchiya et al.(28) | 1 | 20 | F | MELAS |  |  |  |  |  |
| Aharoni et al.(29) | 4* | 52 | F | MELAS |  | + | + |  |  |
|  | -> 3 | <18 |  |  |  |  |  |  |  |
| Kaufman et al.(30) | 1 | 39 | M | MELAS |  | + | + | + | + |
| Köller et al.(31) | 1 | 37 | M | MELAS |  |  | + |  |  |
| Apostolova et al.(32) | 1 | 58 | F | MELAS |  |  | + | + |  |
| Ducreux et al.(33) | 1 | 23 | M | MELAS |  |  |  |  |  |
| Chu et al.(34) | 1 | 30 | M | MELAS |  |  |  |  |  |
| De Luca et al.(35) | 1 | 29 | F | MELAS |  |  |  |  |  |
| Marques-Matos et al.(36) | 1 | 50 | M | MELAS |  |  |  |  |  |
| Seyama et al.(37) | 1 | 29 | M | MELAS |  |  |  |  |  |
| Rusanen et al.(38) | 1 | 38 | M | 3243A>G |  |  |  |  |  |
| Dai et al.(39) | 1 | 37 | M | 3243A>G |  |  |  |  |  |
| Pröbstel et al.(40) | 1 | 60 | M | 3243A>G |  |  |  |  |  |
| Dickerson et al.(41) | 1 | 61 | F | 13513G>A MELAS |  | + |  |  |  |
| Lindberg et al.(42) | 1 | 44 | F | 7512T>C MELAS |  |  |  |  |  |
| Connolly et al.(43) | 27* | 4-47 |  | 3260A>G MELAS |  | + |  | + |  |
| Wang et al.(44) | 3 | 22 | F | 13513G>A MELAS/LS |  |  |  |  |  |
|  |  | 16 | M |  |  |  |  |  | + |
|  |  | 11 | F |  |  |  | + |  |  |
| van den Ouweland et al.(45) | 11* | 19-58 |  | 3243A>G MIDD |  |  |  |  |  |
| Chen et al.(46) | 1 | 48 | F | MIDD |  |  |  |  |  |
| Lien et al.(47) | 6* | 8-74 |  | MIDD |  |  |  |  |  |
| Kobayashi et al.(48) | 2* | 41-67 |  | 3243A>G MIDD |  |  |  |  |  |
| Herrero-Martin et al.(49) | 1 | 50 | F | 5521G>A MELAS/ MERRF |  |  |  |  | + |
| Huang et al.(50) | 8 | 19-50 |  | MELAS/ MERRF |  |  |  |  |  |
| Han et al.^49^ | 2* | 38 | F | 8344A>G |  |  |  |  |  |
|  |  | 42 | F |  |  |  |  |  |  |
| Larsson et al.(51) | 2 | 21 | M | MERRF |  |  | + |  |  |
|  |  | 20 | M |  |  |  |  |  |  |
| Teive et al.(52) | 1 | 52 | M | MERRF |  |  |  |  |  |
| Taylor et al.(53) | 1 | 29 | F | MERRF |  |  |  |  |  |
| Mancuso et al.(54) | 1 | 42 | F | 611G>A MERRF |  |  |  |  |  |
| Young et al.(55) | 1 | 57 | F | 586G>A |  |  |  |  | + |
| Morten et al.(56) | 1 | 31 | F | 3252A>G |  |  |  |  |  |
| Amemiya et al.(57) | 1 | 29 | M+ | 3256C>T |  | + |  |  | + |
| Jaksch et al.(58) | 1 | 33 | M | 3274A>G |  |  | + |  | + |
| Silvestri et al.(59) | 1 | 36 | F | 5540G>A |  |  |  |  |  |
| Nelson et al.(60) | 1 | 45 | M | 5549G>A |  |  |  |  | + |
| Djordjevic et al.(61) | 1 | 24 | F | 5577C>T |  | + | + |  |  |
| Scuderi et al.(62) | 1 | 30 | F | 5814A>G |  |  |  |  | + |
| Bidooki et al.(63) | 1 | 36 | F | 7480A>G |  |  |  |  |  |
| Koubeissi et al.(64) | 1 | 42 | F | 8296A>G |  |  |  |  |  |
| Houshmand et al.(65) | 1 | 48 | F | 8328G>A |  |  |  |  |  |
| Sano et al.(66) | 1 | 31 | M | 8356T>C |  |  |  |  |  |
| Hanna et al.(67) | 1 | 36 | F | 9952G>A |  | + |  |  |  |
| Mezuki et al.(68) | 1 | 55 | M | 10158T>C ND3 |  |  |  |  |  |
| Taylor et al.(69) | 1 | 42 | M | 10191T>C in ND3 |  |  |  |  |  |
| Deschauer et al.(70) | 1 | 67 | M | 11777C>A in ND4 |  | + |  |  |  |
| Coku et al.(71) | 1 | 35 | F | 12276G>A | + |  |  |  |  |
| Slawek et al.(72) | 1 | 21 | M | 13042G>A |  |  |  |  |  |
| Schinwelski et al.(73) | 1 | 26 | M | 13042G>A |  |  |  |  |  |
| Sikorska et al.(74) | 17* |  |  | 4052T>C & 9035T>C |  |  |  |  |  |
| Corona et al.(75) | 4* | 27-64 |  | 4284G>A |  |  |  |  | + |
| Santorelli et al.(76) | 12* | 16-70 |  | 8363G>A |  |  |  |  |  |
|  | 7* | 24-44 |  |  |  |  |  | + |  |
| Virgilio et al.(77) | 7* | 29-65 |  | 8363G>A |  |  |  |  | + |
| Tsao et al.(78) | 14* | 6-58 |  | 8993T>G |  |  |  |  | + |
| Arenas et al.(79) | 5* | 27-62 |  | 8296A>G & 8363G>A |  | + |  |  |  |
| Wei & Wang(80) | 1 | 26 | M | m.9176T>C |  |  |  |  |  |
| Shoffner et al.(81) | 1 | 28 | M | tRNA deletion 3271-3273 |  | + |  |  |  |
| Debray et al.(82) | 1 | 18 | F | 7402delC |  |  |  |  |  |
| De Coo et al.(83) | 1 | 20 | M | 4bp deletion at 14787 |  |  | + | + | + |
| Nishihara et al.(84) | 1 | 60 | F | 2bp deletion in C12orf65 |  |  |  |  |  |
| Puoti et al.(85) | 1 | 48 | F | KSS |  |  |  |  |  |
| Van Goethem et al.(86) | 1 | 18 | M | *POLG* |  |  |  |  |  |
| Mancuso et al.(87) | 1 | 48 | M | *POLG* |  |  |  |  |  |
| Luoma et al.(88) | 1 | 44 | F | *POLG* |  |  | + |  | + |
| Deschauer et al.(89) | 1 | 28 | M | *POLG1* |  |  | + |  |  |
| Van Hove et al.(90) | 1 | 71 | F | *POLG* |  |  |  |  | + |
| Martikainen et al.(91) | 1 | 64 | F | c.2993C>T & c.3550G>C in *POLG1* |  | + | + |  |  |
| Synofzik et al.(92) | 2* | 32 | F | W748S in *POLG* |  |  |  |  |  |
|  |  | 40 | F |  |  |  |  |  |  |
| Hakonen et al.(93) | 2 | 31 | F | W748S & E1143G in *POLG* |  | + | + |  | + |
|  |  | 57 | M |  |  |  |  |  | + |
| Hansen et al.(94) | 1 | 23 | F | p.A467T & p.W748S in *POLG1* |  |  |  |  |  |
| Hudson et al.(95) | 6* | 41-74 |  | *POLG1* |  |  |  |  |  |
| Melberg et al.(96) | 2* | 60-61 |  | *POLG1* |  |  |  |  |  |
| Bee et al.(97) | 2 | 48 | M | c.673C>T |  |  | + |  |  |
|  |  | 48 | M |  |  |  | + |  |  |
|  | 3* | 78-86 |  | c.2447G>A (p.R722H) in *POLG1* |  |  |  |  | + |
| Komulainen et al.(98) | 2* | 17-22 |  |  |  |  |  |  | + |
| Rantamäki et al.(99) | 41* | 19-84 |  | W748S & A467T in *POLG* |  |  |  |  | + |
| Echaniz-Laguna et al.(100) | 2* | 81-82 |  | R374W in *PEO1* |  |  |  |  |  |
|  | 3* | 42-64 |  |  |  |  |  |  |  |
| Gebus et al.(101) | 3* | NK | M | W748S & R627Q in *POLG* |  |  |  |  |  |
|  |  | 58 | M |  |  |  |  |  |  |
|  |  | NK | F |  |  |  |  |  |  |
| Bianco et al.(102) | 1 | 24 | M | m.3460G>A |  |  |  |  |  |
| Morimoto et al.(103) | 1 | 37 | F | LHON |  |  | + |  |  |
| Hirano et al.(104) | 1 | 40 | M | MNGIE |  |  |  |  |  |
| Carod-Artal et al.(105) | 1 | 35 | M | MNGIE |  |  |  |  |  |
| Martí et al.(106) | 1 | 61 | F | MNGIE |  |  |  | + |  |
| Bariş et al.(107) | 1 | 18 | F | MNGIE |  | + |  |  |  |
| Spiegel et al.(108) | 4* | 7-20 |  | MNGIE |  |  | + |  |  |
| Schuepbach et al.(109) | 3* | 20-22 |  | MNGIE |  |  |  |  |  |
| Blondon et al.(110) | 3 |  |  | MNGIE |  |  |  |  |  |
| Massa et al.(111) | 1 | 67 | F | MNGIE |  |  |  |  |  |

**REFERENCES**

1. Morgan-Hughes JA, Hayes DJ, Clark JB, Landon DN, Swash M, Stark RJ, et al. Mitochondrial encephalomyopathies. Biochemical studies in two cases revealing defects in the respiratory chain. Brain. 1982;105:553-82.

2. Suzuki T, Koizumi J, Shiraishi H, Ofuku K, Sasaki M, Hori T, et al. Psychiatric disturbance in mitochondrial encephalomyopathy. Journal of Neurology, Neurosurgery & Psychiatry. 1989;52:920-2.

3. Finsterer J, Stollberger C, Ostermann E, Zuntner G, Huber J, Tscherney R. Recurrent posterior reversible encephalopathy syndrome in mitochondrial disorder. Blood Pressure. 2009;18(3):126-9.

4. Lewandowska E, Schmidt-Sidor B, Mierzewska H, Pasennik E, Kohutnicka M. Ultrastructural study of mother and daughter muscle changes with mitochondrial encephalomyopathy. Folia Neuropathologica. 2001;39(4):271-6.

5. Gopal K, Anand D. Mitochondrial cardiomyopathy: A rare entity. Heart Lung and Circulation. 2017;26 (Supplement 2):S108-S9.

6. Holliday PL, Climie AR, Gilroy J, Mahmud MZ. Mitochondrial myopathy and encephalopathy: three cases--a deficiency of NADH-CoQ dehydrogenase? Neurology. 1983;33(12):1619-22.

7. Majamaa K, Turkka J, Kärppä M, Winqvist S, Hassinen IE. The common MELAS mutation A3243G in mitochondrial DNA among young patients with an occipital brain infarct. Neurology. 1997;49:1331-4.

8. Penn AMW, Lee JWK, Thuillier P, Wagner M, Maclure KM, Menard MR, et al. MELAS sindrome with mitochondrial tRNALeu(UUR) mutation: Correlation of clinical stat, nerve conduction, and muscle 31P magnetic resonance spectroscopy during treatment with nicotinamide and riboflavin. Neurology. 1992;42:2147-52.

9. Onishi H, Inoue K, Osaka H, Kimura S, Nagatomo H, Hanihara T, et al. Mitochondrial myopathy, encephalopathy, lactic acidosis and stroke-like episodes (MELAS) and diabetes mellitus: molecular genetic analysis and family study. Journal of the Neurological Sciences. 1993;114:205-8.

10. Kishimoto M, Hashiramoto M, Kanda F, Tanaka M, Kasuga M. Mitochondrial mutation in diabetic patient with gastrointestinal symptoms [9]. Lancet. 1995;345(8947):452.

11. Gilchrist JM, Sikirica M, Stopa E, Shanske S. Adult-onset MELAS - Evidence for involvement of neurons as well as cerebral vasculature in strokelike episodes. Stroke. 1996;27(8):1420-3.

12. Di Trapani G, Gregori B, Servidei S, Ricci E, Sabatelli M, Tonali P. Mitochondrial encephalopathy, lactic acidosis, and stroke-like episodes (MELAS). Clinical Neuropathology. 1997;16(4):195-200.

13. Huang CN, Jee SH, Hwang JJ, Kuo YF, Chuang LM. Autoimmune IDDM in a sporadic MELAS patient with mitochondrial tRNA(Leu(UUR)) mutation. Clinical Endocrinology. 1998;49(2):265-70.

14. Kimata KG, Gordan L, Ajax ET, Davis PH, Grabowski T. A case of late-onset MELAS. Archives of Neurology. 1998;55(5):722-5.

15. Sharfstein SR, Gordon MF, Libman RD, Malkin ES. Adult-onset MELAS presenting as herpes encephalitis. Archives of Neurology. 1999;56(2):241-3.

16. Silvestri G, Rana M, Odoardi F, Modoni A, Paris E, Papacci M, et al. Single-fiber PCR in MELAS(3243) patients: correlations between intratissue distribution and phenotypic expression of the mtDNA(A3243G) genotype. American Journal of Medical Genetics. 2000;94(3):201-6.

17. Feddersen B, Bender A, Arnold S, Klopstock T, Noachtar S. Aggressive confusional state as a clinical manifestation of status epilepticus in MELAS. Neurology. 2003;61(8):1149-50.

18. Conway LJ, Robertson TE, McGill JJ, Hanson JP. MELAS syndrome in an Indigenous Australian woman. Medical Journal of Australia. 2011;195(10):581-2.

19. Emmanuele V, Garcia-Cazorla A, Huang HB, Coku J, Dorado B, Cortes EP, et al. Decreased hippocampal expression of calbindin D28K and cognitive impairment in MELAS. Journal of the Neurological Sciences. 2012;317(1-2):29-34.

20. Benninger F, Shemesh T, Steiner I. Acute confusion and seizures in a 63-year-old woman. Journal of Clinical Neuroscience. 2013;20(1):139+89.

21. Collorone S, Pontecorvo S, Francia A. Adult-onset of mitochondrial encephalomyopathy lactic acidosis and stroke-like episodes (MELAS) syndrome mimicking herpes encephalitis: A case report and review of literature. Journal of the Neurological Sciences. 2013;1):e595.

22. Prasad M, Narayan B, Prasad AN, Rupar CA, Levin S, Kronick J, et al. MELAS: A multigenerational impact of the MTTL1 A3243G MELAS mutation. Canadian Journal of Neurological Sciences. 2014;41(2):210-9.

23. Sparaco M, Simonati A, Cavallaro T, Bartolomei L, Grauso M, Piscioli F, et al. MELAS: Clinical phenotype and morphological brain abnormalities. Acta Neuropathologica. 2003;106(3):202-12.

24. Dubeau F, De Stefano N, Zifkin BG, Arnold DL, Shoubridge EA. Oxidative phosphorylation defect in the brains of carriers of the tRNAleu(UUR) A3243G mutation in a MELAS pedigree. Ann Neurol. 2000;47(2):179-85.

25. Fang GL, Zheng Y, Zhang YX. Mitochondrial encephalomyopathy with lactic acidosis and stroke-like episodes in an older adult mimicking cerebral infarction: A chinese case report. Clinical interventions in aging. 2018;13:2421-4.

26. Smith K, Chiu S, Hunt C, Chandregowda A, Babovic-Vuksanovic D, Keegan BM. Late-onset Mitochondrial Encephalopathy, Lactic Acidosis, and Stroke-like Episodes Presenting with Auditory Agnosia. Neurologist. 2019;24(3):90-2.

27. Isozumi K, Fukuuchi Y, Tanaka K, Nogawa S, Ishihara T, Sakuta R. A MELAS (mitochondrial myopathy, encephalopathy, lactic acidosis, and stroke-like episodes) mtDNA mutation that induces subacute dementia which mimicks Creutzfeldt-Jakob disease. Internal Medicine. 1994;33(9):543-6.

28. Tsuchiya K, Miyazaki H, Akabane H, Yamamoto M, Kondo H, Mizusawa H, et al. MELAS with prominent white matter gliosis and atrophy of the cerebellar granular layer: A clinical, genetic, and pathological study. Acta Neuropathologica. 1999;97(5):520-4.

29. Aharoni S, Traves TA, Melamed E, Cohen S, Silver EL. MELAS syndrome associated with both A3243G-tRNALeu mutation and multiple mitochondrial DNA deletions. Journal of the Neurological Sciences. 2010;296(1-2):101-3.

30. Kaufman KR, Zuber N, Rueda-Lara MA, Tobia A. MELAS with recurrent complex partial seizures, nonconvulsive status epilepticus, psychosis, and behavioral disturbances: Case analysis with literature review. Epilepsy and Behavior. 2010;18(4):494-7.

31. Köller H, Kornischka J, Neuen-Jacob E, Saleh A, von Giesen H-J, Schmiedel J, et al. Persistent organic personality change as rare psychiatric manifestation of MELAS syndrome. Journal of Neurology. 2003;250:1501-2.

32. Apostolova LG, White M, Moore SA, Davis PH. Deep White Matter Pathologic Features in Watershed Regions. Archives of Neurology. 2005;62:1154-6.

33. Ducreux D, Nasser G, Lacroix C, Adams D, Lasjaunias P. MR diffusion tensor imaging, fiber tracking, and single-voxel spectroscopy findings in an unusual MELAS case. Ajnr: American Journal of Neuroradiology. 2005;26(7):1840-4.

34. Chu CS, Chu CL, Liu HE, Lu T. Regain of visuospatial capacity after coenzyme Q10 in a patient with mitochondrial myopathy, encephalopathy, lactic acidosis and stroke-like episodes: A case report. Acta Neuropsychiatrica. 2012;24(3):186-8.

35. De Luca R, Russo M, Leonardi S, Spadaro L, Cicero C, Naro A, et al. Advances in the treatment of MELAS syndrome: Could cognitive rehabilitation have a role? Applied Neuropsychology: Adult. 2016;23(1):61-4.

36. Marques-Matos C, Reis J, Reis C, Castro L, Carvalho M. Mitochondrial encephalomyopathy with lactic acidosis and strokelike episodes presenting before 50 years of age: When a stroke is not just a stroke. JAMA Neurology. 2016;73(5):604-6.

37. Seyama K, Suzuki K, Mizuno Y, Yoshida M, Tanaka M, Ozawa T. Mitochondrial encephalomyopathy with lactic acidosis and stroke-like episodes with special reference to the mechanism of cerebral manifestations. Acta Neurol Scand. 1989;80(6):561-8.

38. Rusanen H, Majamaa K, Tolonen U, Remes AM, Myllyla R, Hassinen IE. Demyelinating polyneuropathy in a patient with the tRNA(Leu(UUR)) mutation at base pair 3243 of the mitochondrial DNA. Neurology. 1995;45(6):1188-92.

39. Dai Z-j, Wu C-m, Qian Y-y, Jin J, Wang L, Ruan L-y. Severe atrophy of the cerebellum on magnetic resonance imaging in a Chinese patient with maternally inherited diabetes and deafness with the A3243G mitochondrial DNA mutation. International Journal of Diabetes in Developing Countries. 2015;35(4):588-92.

40. Pröbstel AK, Schaller A, Lieb J, Hench J, Frank S, Fuhr P, et al. Mitochondrial cytopathy with common melas mutation presenting as multiple system atrophy mimic. Neurology: Genetics. 2016;2 (6) (no pagination)(e121).

41. Dickerson BC, Holtzman D, Grant PE, Tian D. Case records of the Massachusetts General Hospital. Case 36-2005. A 61-year-old woman with seizure, disturbed gait, and altered mental status. The New England Journal of Medicine. 2005;353:2271-80.

42. Lindberg C, Moslemi AR, Oldfors A. MELAS syndrome in a patient with a point mutation in MTTS1. Acta Neurologica Scandinavica. 2008;117(2):128-32.

43. Connolly BS, Feigenbaum ASJ, Robinson BH, Dipchand AI, Simon DK, Tarnopolsky MA. MELAS syndrome, cardiomyopathy, rhabdomyolysis, and autism associated with the A3260G mitochondrial DNA mutation. Biochemical and Biophysical Research Communications. 2010;402(2):443-7.

44. Wang Z, Qi XK, Yao S, Chen B, Luan X, Zhang W, et al. Phenotypic patterns of MELAS/LS overlap syndrome associated with m.13513G>A mutation, and neuropathological findings in one autopsy case. Neuropathology. 2010;30(6):606-14.

45. van den Ouweland JMW, Lemkes HHPJ, Ruitenbeek W, Sandkuijl LA, de Vijlder MF, Struyvenberg PAA, et al. Mutation in mitochondrial tRNALeu(UUR) gene in a large pedigree with maternally transmitted type II diabetes mellitus and deafness. Nature Genetics. 1992;1:368-71.

46. Chen Y-N, Liou C-W, Huang C-C, Lin T-K, Wei Y-H. Maternally Inherited Diabetes and Deafness (MIDD) Syndrome: A Clinical and Molecular Genetic Study of a Taiwanese Family. Chang Gung Medical Journal. 2004;27:66-73.

47. Lien L-M, Lee H-C, Wang K-L, Chiu J-C, Chiu H-C, Y-H W. Involvement of nervous system in maternally inherited diabetes and deafness (MIDD) with the A3243G mutation of mitochondrial DNA. Acta Neurologica Scandinavica. 2001;103:159-65.

48. Kobayashi Z, Tsunemi T, Miake H, Tanaka S, Watabiki S., Morokuma Y. A Mother and a Child with Maternally Inherited Diabetes and Deafness (MIDD) Showing Atrophy of the Cerebrum, Cerebellum and Brainstem on Magnetic Resonance Imaging (MRI). Internal Medicine. 2005;44(4):328-31.

49. Herrero-Martin NMD, Ayuso T, Tunon MT, Martin MA, Ruiz-Pesini E, Montoya J. A MELAS/MERRF phenotype associated with the mitochondrial DNA 5521G>A mutation. Journal of Neurology, Neurosurgery and Psychiatry. 2010;81(4):471-2.

50. Huang CC, Kuo HC, Chu CC, Liou CW, Ma YS, Wei YH. Clinical phenotype, prognosis and mitochondrial DNA mutation load in mitochondrial encephalomyopathies. Journal of Biomedical Science. 2002;9(5-6):527-33.

51. Larsson N-G, Tulinius MH, Holme E, Oldfors A, Andersen O, Wahistrom J, et al. Segregation and Manifestations of the mtDNA tRNALys A🡪G(8344) Mutation of Myoclonus Epilepsy and Ragged-Red Fibers (MERRF) Syndrome. The American Journal of Human Genetics. 1992;51:1201-12.

52. Teive HAG, Munhoz RP, Muzzio JA, Scola RH, Kay CK, Raskin S, et al. Cerebellar ataxia, myoclonus, cervical lipomas, and MERRF syndrome. Case report. Movement Disorders. 2008;23(8):1191-2.

53. Taylor D, Haynes HR, Graham A, Gerhand S, Kurian KM. A 29-year-old female with progressive myoclonus and cognitive decline. Case reports in neurological medicine. 2013.

54. Mancuso M, Filosto M, Mootha VK, Rocchi A, Pistolesi S, Murri L, et al. A novel mitochondrial tRNA<sup>Phe</sup> mutation causes MERRF syndrome. Neurology. 2004;62 (11):2119-21.

55. Young TM, Blakely EL, Swalwell H, Carter JE, Kartsounis LD, O'Donovan DG, et al. Mitochondrial transfer RNA(Phe) mutation associated with a progressive neurodegenerative disorder characterized by psychiatric disturbance, dementia, and akinesia-rigidity. Archives of Neurology. 2010;67(11):1399-402.

56. Morten KJ, Cooper JM, Brown GK, Lake BD, Pike E, Poulton J. A new point mutation associated with mitochondrial encephalomyopathy. Human Molecular Genetics. 1993;2(12):2081-7.

57. Amemiya S, Hamamoto M, Goto Y, Komaki H, Nishino I, Nonaka I, et al. Psychosis and progressing dementia: presenting features of a mitochondriopathy. Neurology. 2000;55(4):600-1.

58. Jaksch M, Lochmuller H, Schmitt F, Volpel B, Obermaier-Kusser B, Horvath R. A mutation in mt tRNALeu(UUR) causing a neuropsychiatric syndrome with depression and cataract. Neurology. 2001;57(10):1930-1.

59. Silvestri G, Mongini T, Odoardi F, Modoni A, deRosa G, Doriguzzi C, et al. A new mtDNA mutation associated with a progressive encephalopathy and cytochrome c oxidase deficiency. Neurology. 2000;54(8):1693-6.

60. Nelson I, Hanna M, Alsanjari N, Scaravilli F, Morgan-Hughes J, Harding A. A new mitochondrial DNA mutation associated with progressive dementia and chorea: A clinical, pathological, and molecular genetic study. Annals of Neurology. 1995;37(3):400-3.

61. Djordjevic D, Brady L, Bai R, Tarnopolsky MA. Two novel mitochondrial tRNA mutations, A7495G (tRNA(Ser(UCN))) and C5577T (tRNA(Trp)), are associated with seizures and cardiac dysfunction. Mitochondrion. 2016;31:40-4.

62. Scuderi C, Borgione E, Musumeci S, Elia M, Castello F, Fichera M, et al. Severe encephalomyopathy in a patient with homoplasmic A5814G point mutation in mitochondrial tRNACys gene. Neuromuscular Disorders. 2007;3:258-61.

63. Bidooki S, Jackson MJ, Johnson MA, Chrzanowska-Lightowlers ZMA, Taylor RW, Venables G, et al. Sporadic mitochondrial myopathy due to a new mutation in the mitochondrial tRNA<sup>Ser(UCN)</sup> gene. Neuromuscular Disorders. 2004;14(7):417-20.

64. Koubeissi MZ, Khongkhatithum C, Janus AI, Luders H. Scotosensitive myoclonic seizures in MERRF. Neurology. 2009;72(9):858.

65. Houshmand M, Lindberg, C., Moslemi, A-R., Oldfors, A., & Holme, E A novel heteroplasmic point mutation in the mitochondrial tRNALys gene in a sporadic case of mitochondrial encephalomyopathy: De novo mutation and no transmission to the offspring. Human Mutation. 1999;13(3):203-9.

66. Sano M, Ozawa M, Shiota S, Momose Y, Uchigata M, Goto YI. The T-C((8356)) mitochondrial DNA mutation in a Japanese family. Journal of Neurology. 1996;243(6):441-4.

67. Hanna MG, Nelson IP, Rahman S, Lane RJM, Land J, Heales S, et al. Cytochrome c Oxidase Deficiency Associated with the First Stop-Codon Point Mutation in Human mtDNA. The American Journal of Human Genetics. 1998;63:29-36.

68. Mezuki S, Fukuda K, Matsushita T, Fukushima Y, Matsuo R, Goto YI, et al. Isolated and repeated stroke-like episodes in a middle-aged man with a mitochondrial ND3 T10158C mutation: a case report. BMC Neurol. 2017;17(1):217.

69. Taylor RW, Singh-Kler R, Hayes CM, Smith PEM, Turnbull DM. Progressive mitochondrial disease resulting from a novel missense mutation in the mitochondrial DNA ND3 gene. Annals of Neurology. 2001;50(1):104-7.

70. Deschauer M, Bamberg C, Claus D, Zierz S, Turnbull DM, Taylor RW. Late-onset encephalopathy associated with a C11777A mutation of mitochondrial DNA. Neurology. 2003;60:1357-9.

71. Coku J, Shanske S, Mehrazin M, Tanji K, Naini A, Emmanuele V, et al. Slowly progressive encephalopathy with hearing loss due to a mutation in the mtDNA tRNA(Leu(CUN)) gene. Journal of the Neurological Sciences. 2010;290(1-2):166-8.

72. Slawek J, Kierdaszuk B, Tonska K, Kodron A, Schinwelski M, Sitek EJ, et al. Mitochondrial encephalopathy in a patient with a 13042G>A de novo mutation. Journal of Clinical Pathology. 2012;65(12):1147-9.

73. Schinwelski M, Kierdaszuk B, Dulski J, Tonska K, Kodron A, Sitek EJ, et al. Changing phenotypic expression in a patient with a mitochondrial encephalopathy due to 13042G>A de novo mutation-a 5 year follow up. Metabolic Brain Disease. 2015;30(4):1083-5.

74. Sikorska M, Sandhu JK, Simon DK, Pathiraja V, Sodja C, Li Y, et al. Identification of ataxia-associated mtDNA mutations (m.4052T>C and m.9035T>C) and evaluation of their pathogenicity in transmitochondrial cybrids. Muscle & Nerve. 2009;40(3):381-94.

75. Corona P, Lamantea E, Greco M, Carrara F, Agostino A, Guidetti D, et al. Novel heteroplasmic mtDNA mutation in a family with heterogeneous clinical presentations. Annals of Neurology. 2002;51(1):118-22.

76. Santorelli FM, Mak S-C, El-Schahawi M, Casali C, Shanske S, Baram TZ, et al. Maternally Inherited Cardiomyopathy and Hearing Loss Associated with a Novel Mutation in the Mitochondrial tRNA1LY Gene (G8363A). American Journal of Human Genetics. 1996;58:933-9.

77. Virgilio R, Ronchi D, Bordoni A, Fassone E, Bonato S, Donadoni C, et al. Mitochondrial DNA G8363A mutation in the tRNA<sup>Lys</sup> gene: Clinical, biochemical and pathological study. Journal of the Neurological Sciences. 2009;281(1-2):85-92.

78. Tsao CY, Mendell JR, Bartholomew D. High mitochondrial DNA T8993G mutation (<90%) without typical features of Leigh's and NARP syndromes. Journal of Child Neurology. 2001;16(7):533-5.

79. Arenas J, Campos Y, Bornstein B, Ribacoba R, Martin MA, Rubio JC, et al. A double mutation (A8296G and G8363A) in the mitochondrial DNA tRNALys gene associated with myoclonus epilepsy with ragged-red fibers. Neurology. 1999;52(2):377-82.

80. Wei Y, Wang L. Adult-onset Leigh syndrome with central fever and peripheral neuropathy due to mitochondrial 9176T>C mutation. Neurological Sciences. 2018;39(12):2225-8.

81. Shoffner JM, Bialer MG, Pavlakis SG, Lott M, Kaufman A, Dixon J, et al. Mitochondrial encephalomyopathy associated with a single nucleotide pair deletion in the mitochondrial tRNALeu(UUR) gene. Neurology. 1995;45(2):286-92.

82. Debray FG, Seneca S, Gonce M, Vancampenhaut K, Bianchi E, Boemer F, et al. Mitochondrial encephalomyopathy with cytochrome c oxidase deficiency caused by a novel mutation in the MTCO1 gene. Mitochondrion. 2014;17:101-5.

83. De Coo IFM, Renier WO, Ruitenbeek W, Ter Laak HJ, Bakker M, Schägger H, et al. A 4–base pair deletion in the mitochondrial cytochrome b gene associated with parkinsonism/MELAS overlap syndrome. Annals of Neurology. 1999;45(1):130-3.

84. Nishihara H, Omoto M, Takao M, Higuchi Y, Koga M, Kawai M, et al. Autopsy case of the C12orf65 mutation in a patient with signs of mitochondrial dysfunction. Neurology: Genetics. 2017;3(4).

85. Puoti G, Carrara F, Sampaolo S, De Caro M, Vincitorio CM, Invernizzi F, et al. Identical large scale rearrangement of mitochondrial DNA causes Kearns-Sayre syndrome in a mother and her son. Journal of Medical Genetics. 2003;40(11):858-63.

86. Van Goethem G, Mercelis R, Lofgren A, Seneca S, Ceuterick C, Martin JJ, et al. Patient homozygous for a recessive POLG mutation presents with features of MERRF. Neurology. 2003;61(12):1811-3.

87. Mancuso M, Filosto M, Bellan M, Liguori R, Montagna P, Baruzzi A, et al. POLG mutations causing ophthalmoplegia, sensorimotor polyneuropathy, ataxia, and deafness. Neurology. 2004;62(2):316-8.

88. Luoma PT, Luo N, Löscher WN, Farr CL, Horvath R, Wanschitz J, et al. Functional defects due to spacer-region mutations of human mitochondrial DNA polymerase in a family with an ataxia-myopathy syndrome. Human Molecular Genetics. 2005;14(14):1907-20.

89. Deschauer M, Tennant S, Rokicka A, He L, Kraya T, Turnbull DM, et al. MELAS associated with mutations in the POLG1 gene. Neurology. 2007;68(20):1741-2.

90. Van Hove JLK, Cunningham V, Rice C, Ringel SP, Zhang Q, Chou PC, et al. Finding Twinkle in the Eyes of a 71-Year-Old Lady: A Case Report and Review of the Genotypic and Phenotypic Spectrum of TWINKLE-Related Dominant Disease. American Journal of Medical Genetics Part A. 2009;149A(5):861-7.

91. Martikainen MH, Hinttala R, Majamaa K. Novel POLG1 mutations in a patient with adult-onset progressive external ophthalmoplegia and encephalopathy. BMJ Case Rep. 2010;2010.

92. Synofzik M, Schule R, Schulte C, Kruger R, Lindig T, Schols L, et al. Complex hyperkinetic movement disorders associated with POLG mutations. Movement Disorders. 2010;25(14):2472-5.

93. Hakonen AH, Heiskanen S, Juvonen V, Lappalainen I, Luoma PT, Rantamaki M, et al. Mitochondrial DNA polymerase W748S mutation: A common cause of autosomal recessive ataxia with ancient European origin. American Journal of Human Genetics. 2005;77(3):430-41.

94. Hansen N, Zwarg T, Wanke I, Zierz S, Kastrup O, Deschauer M. MELAS/SANDO overlap syndrome associated with POLG1 mutations. Neurological Sciences. 2012;33(1):209-12.

95. Hudson G, Deschauer M, Busse K, Zierz S, Chinnery PF. Sensory ataxic neuropathy due to a novel C10Orf2 mutation with probable germline mosaicism. Neurology. 2005;64(2):371-3.

96. Melberg A, Nennesmo I, Moslemi AR, Kollberg G, Luoma P, Suomalainen A, et al. Alzheimer pathology associated with POLG1 mutation, multiple mtDNA deletions, and APOE4/4: Premature ageing or just coincidence? [2]. Acta Neuropathologica. 2005;110(3):315-6.

97. Bee L, Nasca A, Zanolini A, Cendron F, d'Adamo P, Costa R, et al. A nonsense mutation of human XRCC4 is associated with adult-onset progressive encephalocardiomyopathy. EMBO Mol Med. 2015;7(7):918-29.

98. Komulainen T, Hinttala R, Karppa M, Pajunen L, Finnila S, Tuominen H, et al. POLG1 p.R722H mutation associated with multiple mtDNA deletions and a neurological phenotype. BMC Neurology. 2010;10 (no pagination)(29).

99. Rantamäki M, Luoma P, Virta JJ, Rinne JO, Paetau A, Suomalainen A, et al. Do carriers of POLG mutation W748S have disease manifestations? Clinical Genetics. 2007;72(6):532-7.

100. Echaniz-Laguna A, Chanson JB, Wilhelm JM, Sellal F, Mayencon M, Mohr M, et al. A novel variation in the Twinkle linker region causing late-onset dementia. Neurogenetics. 2010;11(1):21-5.

101. Gebus O, Fleury M, Chanson JB, Anheim M, Tranchant C, Echaniz-Laguna A. Mitochondrial disease and amyloidosis in a patient with familial polyneuropathy. European Journal of Neurology. 2018;25(11):e118-e9.

102. Bianco A, Bisceglia L, De Caro MF, Galeandro V, De Bonis P, Tullo A, et al. Leber's hereditary optic neuropathy, intellectual disability and epilepsy presenting with variable penetrance associated to the m.3460G>A mutation and a heteroplasmic expansion of the microsatellite in MTRNR1 gene - case report. BMC Medical Genetics. 2018;19 (1) (no pagination)(129).

103. Morimoto N, Nagano I, Deguchi K, Murakami T, Fushimi S, Shoji M, et al. Leber hereditary optic neuropathy with chorea and dementia resembling Huntington disease. Neurology. 2004;63(12):2451-2.

104. Hirano M, Silvestri G, Blake DM, Lombes A, Minetti C, Bonilla E, et al. Mitochondrial neurogastrointestinal encephalomyopathy (MNGIE): Clinical, biochemical, and genetic features of an autosomal recessive mitochondrial disorder. Neurology. 1994;44:721-7.

105. Carod-Artal FJ, Herrero MD, Lara MC, Lopez-Gallardo E, Ruiz-Pesini E, Marti R, et al. Cognitive dysfunction and hypogonadotrophic hypogonadism in a Brazilian patient with mitochondrial neurogastrointestinal encephalomyopathy and a novel ECGF1 mutation. European Journal of Neurology. 2007;14(5):581-5.

106. Martí R, Verschuuren JJ, Buchman A, Hirano I, Tadesse S, van Kuilenburg AB, et al. Late-onset MNGIE due to partial loss of thymidine phosphorylase activity. Annals of Neurology. 2005;58(4):649-52.

107. Bariş Z, Eminoğlu T, Dalgiç B, Tümer L, Hasanoğlu A. Mitochondrial neurogastrointestinal encephalomyopathy (MNGIE): case report with a new mutation. European Journal of Pediatrics. 2010;169(11):1375-8.

108. Spiegel R, Shaag A, Edvardson S, Mandel H, Stepensky P, Shalev SA, et al. SLC25A19 Mutation as a Cause of Neuropathy and Bilateral Striatal Necrosis. Annals of Neurology. 2009;66(3):419-24.

109. Schupbach WMM, Vadday KM, Schaller A, Brekenfeld C, Kappeler L, Benoist JF, et al. Mitochondrial neurogastrointestinal encephalomyopathy in three siblings: Clinical, genetic and neuroradiological features. Journal of Neurology. 2007;254(2):146-53.

110. Blondon H, Polivka M, Joly F, Flourie B, Mikol J, Messing B. Digestive smooth muscle mitochondrial myopathy in patients with mitochondrial-neuro-gastro-intestinal encephalomyopathy (MNGIE). Gastroentérologie Clinique et Biologique. 2005;29(8-9):773-8.

111. Massa R, Tessa A, Margollicci M, Micheli V, Romigi A, Tozzi G, et al. Late-onset MNGIE without peripheral neuropathy due to incomplete loss of thymidine phosphorylase activity. Neuromuscular Disorders. 2009;19 (12):837-40.
