# Supplementary material for: Systematic review of cognitive deficits in adult mitochondrial disease
Source: Eur J Neurol. 2019 Oct 22;27(1):3–17. doi: 10.1111/ene.14068 (PMC6916601; doi:10.1111/ene.14068)
Supplement: Supplementary file 3 — Appendix S1. References. [file ENE-27-3-s003.docx]

**REFERENCE LIST FOR TABLE 3**

Goto Y, Horai S, Matsuoka T, Koga Y, Nihei K, Kobayashi M, et al. Mitochondrial myopathy, encephalopathy, lactic acidosis, and stroke-like episodes (MELAS): A correlative study of the clinical features and mitochondrial DNA mutation. Neurology. 1992;42:545-50.

Hirano M, Ricci E, Koenigsberger MR, Defendini R, Pavlakis SG, DeVivo DC, et al. Melas: An orig.Damian MS, Seibel P, Reichmann H, Schachenmayr W, Laube H, Bachmann G, et al. Clinical spectrum of the MELAS mutation in a large pedigree. Acta Neurologica Scandinavica. 1995;92 (5):409-15..Chinnery PF, Howell N, Lightowlers RN, Turnbull DM. Molecular pathology of MELAS and MERRF The relationship between mutation load and clinical phenotypes. Brain. 1997;120:1713-21.

Majamaa K, Moilanen JS, Uimonen S, Remes AM, Salmela PI, Karppa M, et al. Epidemiology of A3243G, the mutation for mitochondrial encephalomyopathy, lactic acidosis, and strokelike episodes: Prevalence of the mutation in an adult population. American Journal of Human Genetics. 1998;63(2):447-54.

Suzuki S, Oka Y, Kadowaki T, Kanatsuka A, Kuzuya T, Kobayashi M, et al. Clinical features of diabetes mellitus with the mitochondrial DNA 3243 (A-G) mutation in Japanese: Maternal inheritance and mitochondria-related complications. Diabetes Research and Clinical Practice. 2003;59 (3):207-17.

Murakami T, Shinoto Y, Yonemitsu S, Muro S, Oki S, Koga Y, et al. Early Onset of Diabetes Mellitus Accelerates Cognitive Decline in Japanese Patients with Mitochondrial Myopathy, Encephalopathy, Lactic Acidosis, and Stroke-Like Episodes. Tohoku J Exp Med. 2016;238(4):311-6.

Chae JH, Hwang H, Lim BC, Cheong HI, Hwang YS, Kim KJ. Clinical features of A3243G mitochondrial tRNA mutation. Brain & Development. 2004;26:459-62.

Sproule DM, Kaufmann P. Mitochondrial Encephalopathy, Lactic Acidosis, and Strokelike Episodes Basic Concepts, Clinical Phenotype, and Therapeutic Management of MELAS Syndrome. Year in Neurology 2008. 2008;1142:133-58.

Lorenzoni PJ, Scola RH, Kamoi Kay CS, Arndt RC, Freund AA, Bruck I, et al. MELAS: Clinical features, muscle biopsy and molecular genetics. . Arquivos de Neuro-Psiquiatria. 2009;67 (3 A):668-76.

Ma WW, Yuan LH, Yu HL, Ding BJ, Xi YD, Feng JF, et al. Genistein as a neuroprotective antioxidant attenuates redox imbalance induced by beta-amyloid peptides 25-35 in PC12 cells. International Journal of Developmental Neuroscience. 2010;28(4):289-95..Hammans SR, Sweeney MG, Brockington M, Lennox GG, Lawton NF, Kennedy CR, et al. The mitochondrial DNA transfer RNA(Lys) A -> G(<sup>8344</sup>) mutation and the syndrome of myoclonic epilepsy with ragged red fibres (MERRF). Relationship of clinical phenotype to proportion of mutant mitochondrial DNA. Brain. 1993;116 (3):617-32.

Ozawa M, Goto YI, Sakuta R, Tanno Y, Tsuji S, Nonaka I. The 8,344 mutation in mitochondrial DNA: A comparison between the proportion of mutant DNA and clinicopathologic findings. Neuromuscular Disorders. 1995;5 (6):483-8.

Sinha S, Satishchandra P, Gayathri N, Yasha TC, Shankar SK. Progressive myoclonic epilepsy: A clinical, electrophysiological and pathological study from South India. Journal of the Neurological Sciences. 2007;252(1):16-23.

Lorenzoni PJ, Scola RH, Kay CK, Arndt RC, Silvado CE, Werneck LC. MERRF: Clinical features, muscle biopsy and molecular genetics in Brazilian patients. Mitochondrion. 2011;11:528-32.

Mancuso M, Orsucci D, Angelini C, Bertini E, Carelli V, Comi GP, et al. Phenotypic heterogeneity of the 8344A>G mtDNA "MERRF" mutation. Neurology. 2013;80(22):2049-54.

Pavlakis SG, Phillips PC, Dimauro S, Devivo DC, Rowland LP. Mitochondrial myopathy, encephalopathy, lactic-acidosis, and strokelike episodes - A distinctive clinical syndrome. Annals of Neurology. 1984;16(4):481-8.

Khambatta S, Nguyen DL, Beckman TJ, Wittich CM. Kearns-Sayre syndrome: A case series of 35 adults and children. International Journal of General Medicine. 2014;7:325-32.

Wray SH, Provenzale JM, Johns DR, Thulborn KR. MR of the brain in mitochondrial myopathy. American Journal of Neuroradiology. 1995;16(5):1167-73.

Van Goethem G, Luoma P, Rantamaki M, Al Memar A, Kaakkola S, Hackman P, et al. POLG mutations in neurodegenerative disorders with ataxia but no muscle involvement. Neurology. 2004;63(7):1251-7.

Winterthun S, Ferrari G, He L, Taylor RW, Zeviani M, Turnbull DM, et al. Autosomal recessive mitochondrial ataxic syndrome due to mitochondrial polymerase gamma mutations. Neurology. 2005;64(7):1204-8.

Hakonen AH, Heiskanen S, Juvonen V, Lappalainen I, Luoma PT, Rantamaki M, et al. Mitochondrial DNA polymerase W748S mutation: A common cause of autosomal recessive ataxia with ancient European origin. American Journal of Human Genetics. 2005;77(3):430-41.

Horvath R, Hudson G, Ferrari G, Fütterer N, Ahola S, Lamantea E, et al. Phenotypic spectrum associated with mutations of the mitochondrial polymerase y gene. Brain. 2006;129:1674-84.

Tzoulis C, Engelsen BA, Telstad W, Aasly J, Zeviani M, Winterthun S, et al. The spectrum of clinical disease caused by the A467T and W748S POLG mutations: A study of 26 cases. Brain. 2006;129 (7):1685-92.

Wong LJ, Naviaux RK, Brunetti-Pierri N, Zhang Q, Schmitt ES, Truong C, et al. Molecular and Clinical Genetics of Mitochondrial Diseases Due to POLG Mutations. Human Mutation. 2008;29:E150-E72.

Van Hove JLK, Cunningham V, Rice C, Ringel SP, Zhang Q, Chou PC, et al. Finding Twinkle in the Eyes of a 71-Year-Old Lady: A Case Report and Review of the Genotypic and Phenotypic Spectrum of TWINKLE-Related Dominant Disease. American Journal of Medical Genetics Part A. 2009;149A(5):861-7.

Naïmi M, Bannwarth S, Procaccio V, Pouget J, Desnuelle C, Pellissier JF, et al. Molecular analysis of ANT1, TWINKLE and POLG in patients with multiple deletions or depletion of mitochondrial DNA by a dHPLC-based assay. European Journal of Human Genetics. 2006;14:917-22.

Jaksch M, Klopstock T, Kurlemann G, Dorner M, Hofmann S, Kleinle S, et al. Progressive myoclonus epilepsy and mitochondrial myopathy associated with mutations in the tRNA(Ser(UCN)) gene. Annals of Neurology. 1998;44(4):635-40.
